# Mutational Landscape and Interaction of SARS-CoV-2 with Host Cellular Components

**DOI:** 10.3390/microorganisms9091794

**Published:** 2021-08-24

**Authors:** Mansi Srivastava, Dwight Hall, Okiemute Beatrice Omoru, Hunter Mathias Gill, Sarah Smith, Sarath Chandra Janga

**Affiliations:** 1 Department of BioHealth Informatics, School of Informatics and Computing, Indiana University Purdue University Indianapolis, Informatics and Communications Technology Complex, 535 West Michigan Street, Indianapolis, IN 46202, USA; mansriva@iu.edu (M.S.); dwwhall@iu.edu (D.H.); oomoru@iu.edu (O.B.O.); hungill@iu.edu (H.M.G.); sls23@iu.edu (S.S.); 2 Center for Computational Biology and Bioinformatics, Indiana University School of Medicine, 410 West 10th Street, Indianapolis, IN 46202, USA; 3 Department of Medical and Molecular Genetics, Indiana University School of Medicine, Medical Research and Library Building, 975 West Walnut Street, Indianapolis, IN 46202, USA

**Keywords:** SARS-CoV-2, host interaction, mutation, variants, RNA-binding proteins, structural proteins

## Abstract

The emergence of severe acute respiratory syndrome coronavirus 2 (SARS-CoV-2) and its rapid evolution has led to a global health crisis. Increasing mutations across the SARS-CoV-2 genome have severely impacted the development of effective therapeutics and vaccines to combat the virus. However, the new SARS-CoV-2 variants and their evolutionary characteristics are not fully understood. Host cellular components such as the ACE2 receptor, RNA-binding proteins (RBPs), microRNAs, small nuclear RNA (snRNA), 18s rRNA, and the 7SL RNA component of the signal recognition particle (SRP) interact with various structural and non-structural proteins of the SARS-CoV-2. Several of these viral proteins are currently being examined for designing antiviral therapeutics. In this review, we discuss current advances in our understanding of various host cellular components targeted by the virus during SARS-CoV-2 infection. We also summarize the mutations across the SARS-CoV-2 genome that directs the evolution of new viral strains. Considering coronaviruses are rapidly evolving in humans, this enables them to escape therapeutic therapies and vaccine-induced immunity. In order to understand the virus’s evolution, it is essential to study its mutational patterns and their impact on host cellular machinery. Finally, we present a comprehensive survey of currently available databases and tools to study viral–host interactions that stand as crucial resources for developing novel therapeutic strategies for combating SARS-CoV-2 infection.

## 1. Introduction

The outbreak of coronavirus disease (COVID-19) was caused by severe acute respiratory syndrome coronavirus (SARS-CoV-2). The virus originated in the city of Wuhan, China, around December 2019 [1,2]. Due to the exponential rise in COVID-19 infections across countries, it was proclaimed a pandemic by the World Health Organization (WHO) on 11 March 2020 [3]. COVID-19 has caused a global health crisis, infecting over 191 million individuals with over 4 million deaths as of June 2021 (https://www.worldometers.info/coronavirus/, accessed on 30 June 2021). The past two decades have marked the emergence of coronavirus outbreaks, resulting in deadly pandemics. In 2002, severe acute respiratory syndrome (SARS-CoV) recorded a death rate of 10%, while in 2012, Middle East respiratory syndrome (MERS-CoV) recorded a devastating death rate of 36% [4,5]. Both viral epidemics were caused by zoonotic coronaviruses that were first cultured in 1966 by Tyrell and Bynoe from patients with the flu and common cold [6,7]. Coronaviruses (CoV) are members of the *Coronaviridae* family, a group of enveloped viruses with a positive-sensed, single-stranded RNA genome [8]. The term coronaviruses was assigned to these viruses because of their crown-like morphology, with surface projections resembling solar corona under the electron microscope [9,10,11].

Coronaviruses are classified into four genera (alpha, beta, gamma, and delta) based on the differences in their protein sequences [12]. Among the four coronavirus genera, beta-coronaviruses comprise most human coronaviruses (HCoVs), including the highly pathogenic SARS-CoV, MERS-CoV, HCoVOC43, HCoV-HKU1, and the recently identified SARS-CoV-2 [13]. Phylogenetic evidence from multiple studies supports the evolutionary origin of beta coronaviruses from bats and rodents. In contrast, birds are the source of gamma and delta coronaviruses, which remain non-pathogenic and harbor enormous genetic diversity [14,15]. Beta coronaviruses, including SARS-CoV-2, have crossed the species barriers and emerged as highly pathogenic viruses causing lethal respiratory infections in humans. Evolutionary analysis reveals several conserved genomic features between SARS-CoV-2 and other beta coronaviruses [16,17].

SARS-CoV-2 is among the largest RNA viruses, ranging from 26-32 kilobases, and comprises two large open reading frames (*ORF1a* and *ORF1b*) [10,14]. The two ORFs translate into replicase polypeptides (pp1a and pp1b) that form the non-structural proteins essential for viral replication [18,19,20]. The complete assembly of SARS-CoV-2 is aided by the structural proteins (spike (S), envelope (E), membrane (M), and nucleoprotein (N)). [18]. RNA-dependent RNA polymerase (RdRP) encoded by SARS-CoV-2 assists in viral replication after it enters the host cell and it is also sought as a potential anti-viral drug target [21,22,23].

RNA viruses demonstrate rapid evolution due to a high mutation rate which is a million times higher than the host mutation rate [24,25]. The high mutation rate in SARS-CoV-2 is attributed to the enormous genome variability that enabled it to the escape host immune response and antiviral therapeutics. The evolving nature of SARS-CoV-2 has resulted in several new strains of the virus across the world, including highly infectious B.1.1.7/Alpha (UK), B.1.351/Beta (South Africa), B. 1.1.28/Gamma (Brazil), B.1.617.2/Delta (India), and C.37/Lambda (South America) variants [26,27,28,29,30]. Multiple studies have reported that rapidly evolving new strains of SARS-CoV-2 exhibit decreased susceptibility to antiviral therapeutics and escape neutralization by vaccine-induced humoral immunity in the host [31,32,33]. The evolution has urged for the need to dissect the molecular features in the virus that enhance its infectious capacity and modulates host cells through direct and indirect interactions in various cellular components. Several studies on sequence variation in the SARS-CoV-2 genome have identified an abundance of mutations in the spike protein of the virus that enables its entry into the host cell [34,35,36,37,38,39].

After evading the host, viruses modulate several key regulators and proteins encoded by the host cell to enable its replication and assembly. Multiple lines of evidence show that RNA-binding proteins (RBPs) and microRNAs (miRNA) are two such classes of regulators that are targeted by viral genomes and exhibit altered functions during infection [40,41,42,43]. Since the emergence of SARS-CoV-2, several studies have illustrated the genomic features of the virus that enable it to hijack the cellular machinery and bypass the host antiviral response [44,45]. However, the underlying mechanism by which SARS-CoV-2 interacts with several of the regulatory proteins and miRNAs in the host cell is not well elaborated. Therefore, understanding the dynamics of interaction between the host and SARS-CoV-2 would provide insights into viral pathogenesis and aid in developing new targets for anti-viral drug therapies.

SARS-CoV-2 has ample potential to form RNA secondary structures due to its large genome size, but 97% of the genome has not been discovered. The 5′ UTR in the SARS-CoV-2 genome was recently shown to exhibit a secondary structure illustrating a four-way junction near the start codon [46]. The presence of such stable structures in the viral genome aids in the translation of viral proteins using host ribosomes. Several other groups have elaborated on the structural elements in the SARS-CoV-2 genome, including 5’ UTR, a ribosomal frame-shifting element between Orf1a/b and 3′ UTR [47,48,49]. Focused studies on viral secondary structures will provide a roadmap for the identification of potential interactions with other viral and host proteins, thereby guiding the targeted drug development for pharmaceutical interventions. This review summarizes the recent insights into the SARS-CoV-2 structures and their interactions with host cellular proteins and miRNAs. We also elaborate on mutations across various genomic regions of the virus that impact viral pathogenesis. Lastly, we provide a comprehensive summary of the publicly available tools and resources for exploring the host and viral interactions.

### 1.1. Interaction of SARS-CoV-2 with Host Cellular Components

Our knowledge of SARS-CoV-2 pathogenesis can be improved by a detailed understanding of various interactions between SARS-CoV-2 and its host cell during infection. These interactions are crucial for the survival of the virus in the host cell [50]. Various structural, non-structural, and accessory proteins interact with host cellular components to regulate the biological processes [51]. Several host RNA molecules such as small nuclear RNA (snRNA), 18s rRNA, and the 7SL RNA component of the signal recognition particle (SRP) are shown to interact with SARS-CoV-2. snRNAs mediate pre-mRNAsplicing to form mature mRNA [52].

**Figure 1:**
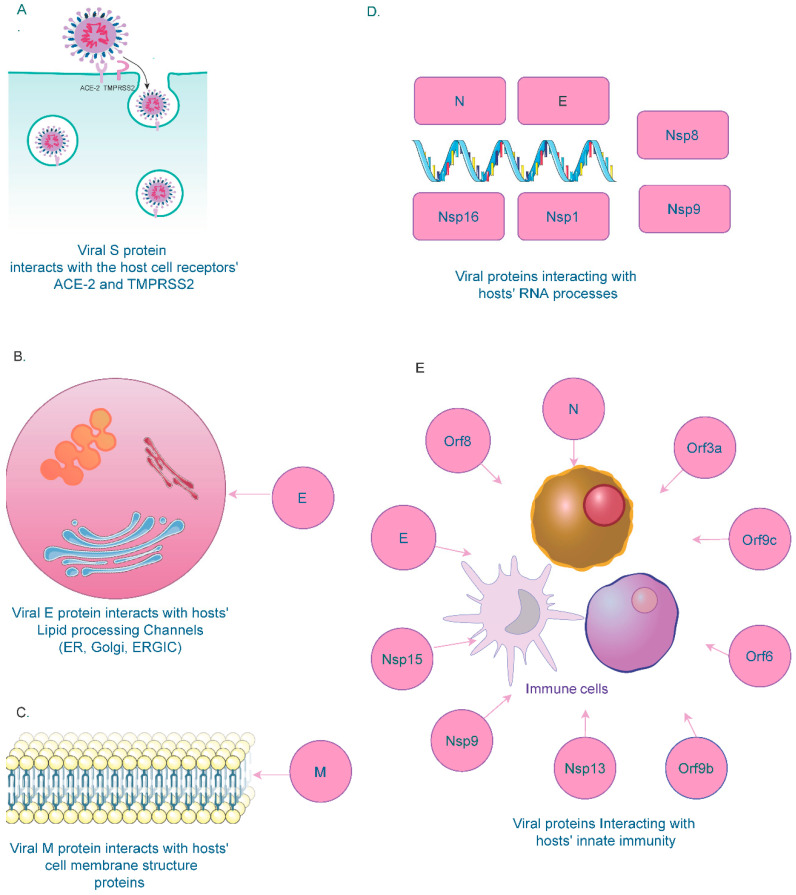
SARS-CoV-2s viral protein interactions with host cell structures and processes. (**A**) SARS-C0V-2 spike (S) protein interacts with host cell receptors ACE2 and TMPRSS2 for cellular entry. (**B**) Host cell membrane structure proteins interact with SARS-CoV-2 membrane (M) proteins for viral morphogenesis. (**C**) Host cell lipid processing channels such as ER, Golgi, and ERGIC interact with the SARS-CoV-2 Envelope (**E**) protein. (**D**) Host RNA processing components interact with SARS-CoV-2 N, E and Nsp (1,8,9,16), encoded by the SARS-CoV-2 genome. (**E**) Several structural and non-structural proteins in the SARS-Co-V-2 genome interact with components of the host innate immune system.

SARS-CoV-2’s spike protein (S) interacts with hosts’ ACE2 and TMPRSS2 receptors for entry (Figure 1A). Studies have shown that interactions between the receptor-binding domain of the spike protein and the ACE2 receptor in the host cell and subsequent proteolytic events drive the fusion of the spike protein with the host cell membrane mediated by TMPRSS2 [53,54].

Studies have shown that the viral Envelope (E) protein is necessary for viral particle formation and binds to BRD2 and BRD4, members of the bromodomain and extra-terminal (BET) domain family proteins in the host [55,56,57]. BETs bind acetylated histones for transcriptional regulation. The C-terminal region of the E protein shares sequence similarity with the N-terminal region of the H3 histone that interacts with bromodomains [58]. Due to this structural similarity, the interaction between the viral E protein and host BRD proteins disrupts the physiological binding of the BRD with histones, thereby altering the host gene expression [59,60]. Due to their role in the viral packaging and assembly, E proteins have been found in the cellular compartments involved in intracellular trafficking, such as the Golgi apparatus, Endoplasmic reticulum (ER), and the ER-Golgi intermediate compartment (ERGIC) [61,62] (Figure 1B). Interactions between the virus and host cellular processes, such as inflammation [63], coagulation [64], autophagy [65], apoptosis [66] and innate immune responses [67], have also been established.

Similar to the E protein, the membrane (M) protein also confines to the ER, Golgi, and ERGIC compartments. The M protein can also be found in the plasma membrane of host cells, in which it interacts with host proteins responsible for the plasma membrane structure (Figure 1C). These proteins include ADD1/2 (alpha and beta-Adducin) [68], EHD1/4 (EH-domain containing proteins) [69], SNX1/2 (Sorting nexin1/2) [70], FCHO2 (F-BAR Domain) [71], and SH3GL3 (Endophilin A3) [72]. Interaction between the viral M protein and host plasma membrane proteins could have favorable functions for viral genome assembly. Another study has demonstrated that the M protein recruits membrane structure components from the host machinery for its morphogenesis [73].

The viral Nucleocapsid (N) protein has various functions in the viral genome. It is responsible for the assembly and encapsulation of the viral genome. Notably, the protein has been identified to interact with various host RNA factors to protect the viral genome from nucleases and pattern-recognition proteins (PRPs), thereby regulating the virion’s transcription, replication, and translation [74].

Among the non-structural proteins, Nsp16 binds to U1 and U2 snRNAs. It attaches to the 5′ splice site of U1 and the branch point site of U2, resulting in the suppression of mRNA splicing in infected cells [75]. Similar to Nsp16, another non-structural protein, Nsp1, binds to host 18s ribosomal RNA that forms the structural component of the 40s ribosomal subunit [76]. This interaction inhibits translation in the host’s mRNA upon SARS-CoV-2 infection. Translation of SARS-CoV-2 RNA is protected from its own Nsp1-mediated translation inhibition by the first stem-loop (SL1) of its 5′ leader sequence [75]. Other host components, such as the signal recognition particle (SRP), play a crucial role during protein synthesis by binding to the 80s ribosome. When the SRP-ribosome complex binds to the SRP receptor, SRP is released from the ribosome to resume protein synthesis, ensuring proper folding and transport to the cell membrane [76]. A recent study demonstrates that Nsp8 and Nsp9 bind the S domain of the 7SL RNA scaffold of SRP. Nsp8 binds to 7SL in the location enclosed by the SRP54 protein, while Nsp9 binds to 7SL in the site surrounded by the SRP19 protein. By binding with 7SL, the viral proteins disrupt SRPs function, thus inhibiting protein trafficking to the cell membrane [75].

Overall, the interaction between SARS-CoV-2 and various host RNA molecules disrupts essential cellular processes in the host, thereby modulating the host cell response during infection (Figure 1D). Overall, several lines of evidence have shown that SARS-CoV-2 interacts with crucial host cell components that are vital for cell survival and homeostasis (Figure 1). However, additional in-depth investigations are required to validate these interactions and enable their use as potential targets for antiviral therapeutics during SARS-CoV-2 infection.

### 1.2. Interaction of SARS-CoV-2 with the Host Immune System

#### 1.2.1. Interaction of SARS-CoV-2 with Host Effector Cell Components

SARS-CoV-2 modulates the host immune response to bypass the antiviral surveillance protective mechanism. Innate immunity plays a central role as the first line of defense to combat viral infection [77,78]. A dysfunctional immune system and lack of appropriate host cell response have been associated with the progression of SARS-CoV-2 infection [79]. SARS-CoV-2 invasion triggers an innate immune response in the infected individuals [80]. Various viral structures (N, E, Nsp9, Nsp13, Nsp15, Orf3a, Orf6, Orf8, Orf9b, and Orf9c) have been identified to interact with several components of the host innate immune system (Figure 1E). During the acute phase of infection, B cells recognize the invading viral antigens that are then presented to T cells by the MHC complex. This process enhances immune cells’ cytotoxic activity, resulting in increased cytokine secretion and antibody production [81,82]. SARS-CoV-2 infections have been primarily associated with deteriorating impacts on upper respiratory airways and the nasal passage [83]. However, the host immune system interaction with SARS-CoV-2 in the nasal barriers and upper airways is not entirely understood. Interaction of SARS-CoV-2 viral genes with specific host immune components is critical in determining the extent of virulence and disease severity [84].

#### 1.2.2. Interaction of SARS-CoV-2 with Host Intracellular Components

Several signaling pathways are also activated during the onset of viral infection. Recent studies show activation of interferon (IFN) signaling upon SARS-CoV-2 infection [85,86]. A study on RNA-protein interactome using a human liver cell line (Huh7) illustrated a significant upregulation of IFN signaling pathways in infected cells [87]. The study’s results confirm that viral infection can trigger the host’s innate immune response by activating the anti-viral signaling pathways. Several of the viral non-structural proteins (Orf6, Orf9, Nsp13, and Nsp15) targets proteins of the IFN pathway, resulting in a dysregulated immune response [77,88,89].

Other immune signaling pathways that the viral proteins have targeted include NF-kb targeted by Nsp13(TLE1, 3, and 5), and Orf9c (NLRX1, F2RL1, NDF1P2) and E3 ubiquitin ligase targeted by Orf3a(TRIM59), N(TRIM26), and Nsp9(MIB1) [90]. Orf10 has been shown to interact with the CUL2^ZYG11B^ complex, a Cullin 2 RING E3 ligase complex member and the complex is comprised of ubiquitin ligases involved in the regulation of a multitude of functions in eukaryotes. Interaction between the Orf10 and CUL2^ZYG11B^ complex has been shown to obstruct the ubiquitination pathway [90,91]. The viral E protein interacts with host TMEM9B, resulting in the activation of NF-kB and MAPK pathways that rapidly enhances the production of inflammatory cytokines such as IL-6, IL-1β, and TNFα [92]. Studies have shown an interaction between Orf8 and IL-17 receptor A (IL-17RA), suggesting that this interaction regulates IL-17 signaling [93] and infection with SARS-CoV-2 virus with deletions of Orf8 associated with milder disease symptoms and low pro-inflammatory cytokine secretions [94]. Figure 1E summarizes the various viral components (structural and non-structural proteins) interacting with the host immune system.

The cellular nucleic acid-binding protein (CNBP) functions as an antiviral regulator essential for innate immune response activation. It is associated with modulating pro-inflammatory cytokines in response to foreign nucleic acid [95]. Studies have shown their significant effect on virus-induced cell death [96]. CNBP knockout cells with SARS-CoV-2 infection showed significantly higher viral titers than matched control cells [87]. A previous study has indicated the interaction between SARS-CoV-2 RNA and evolutionarily conserved RNA-binding protein La-related protein 1 (LARP1) that binds to the 5′ leader sequence of SARS-CoV-2. It was found that depletion of LARP1 in SARS-CoV-2-infected cells resulted in higher viral titers [87]. LARP1 is shown to be the direct substrate for phosphorylation by the mammalian target of the rapamycin complex (mTORC1), which functions as a translation regulator [97]. Previous studies have demonstrated that mTORC1 utilizes LARP1 as a molecular switch to control the translation of several classes of mRNAs including TOP (terminal oligopyrimidine) mRNAs [98,99,100]. Interestingly, the inhibition of mTOR has recently been shown to impede SARS-CoV-2 replication in the human Caco-2 (colon carcinoma) cell line [101,102]. These findings indicate that the interaction between SARS-CoV-2 and the host mTOR protein may have a role in viral protein synthesis. RyDEN, an IFN-induced protein in the host, has been shown to suppress ribosomal frameshifting during SARS-CoV-2 RNA translation [87].

Studies suggest that severe cases of SARS-CoV-2 infection develop a cytokine storm, which is marked by a sudden increase in the level of cytokines during the infection [103]. Host immune cells respond to SARS-CoV-2 infection by causing a hyperactive inflammatory response with the release of pro-inflammatory cytokines such as IL-1, IL-6, and TNFα [104]. Cytokine storm correlates positively with lung injury, multi-organ failure, and poor outcomes of COVID-19 [104,105]. SARS-CoV-2 infection has been associated with a surge in the cytokine storm that leads to acute respiratory distress syndrome (ARDS), which is a major cause of mortality in SARS-CoV-2-infected individuals [106,107,108]. Although the exact cause of ARDS in SARS-CoV-2 is not well established, the increased production of pro-inflammatory cytokines is considered a major contributing factor [103]. A study on cytokine levels in plasma obtained from 41 COVID-19 cases showed increased levels of cytokines including 1L-1β, IL-7, IL-8, IL-9, IL-10, TNF-α, and IFN-γ [103].

Elderly people account for the majority of the fatal cases of COVID-19 reported to date [109]. This extremely high proportion of death can be explained by an increase in NLRP3 inflammasome expression in lungs infected with SARS-CoV-2. NLRP3 inflammasome activation contributes to the lung inflammation and fibrosis seen in infected patients [110]. A recent study has found that SARS-CoV-2-encoded proteases (NSP3/papain-like protease and NSP5/3C-like protease) that cleave polyproteins during viral replication could also play a crucial role in the cleavage of host cellular proteins including IRF3, TAB1, and NLRP12 [111]. This could further result in an impaired type I IFN response and abrupt the production of cytokines from the infected cells.

SARS-CoV-2 infection in a transgenic mice model has been shown to induce inflammatory response, apoptosis, and necroptosis [66]. A recent study has demonstrated that SARS-CoV-2 ORF-3a possesses pro-apoptotic abilities [112]. ORF-3a has been shown to promote the regulated cleavage and activation of caspase 8, which is a known indicator of the extrinsic apoptotic pathway [113]. Caspase 8 activation upregulates the secretion of pro-inflammatory cytokines such as IL-1β. It transforms pro-IL-1β into its mature and bioactive form, which in turn provokes the apoptotic pathway resulting in inflammatory responses [114]. Activation of apoptotic pathways leads to the formation of cellular debris and pulmonary fibrosis that was observed in deceased lungs of fatal COVID-19 cases [115]. Down-regulation of pro-IL-1β secretion has been observed in SARS-CoV-2-infected cells treated with the NF-kB inhibitor, suggesting that SARS-CoV-2-induced inflammatory responses also depend on the NF-kB pathway [66]. Recently, Chu et al. demonstrated that targeted inhibition of intrinsic apoptotic pathways protected SARS-CoV-2-infected mice from severe lung injury [116]. Thus, the results from their study indicated that targeting viral-induced apoptosis could be a potential strategy to combat viral infection and pathogenic damage.

The interaction between CD26/DDP4 (cluster of differentiation-26/T-cell regulatory dipeptidyl peptidase 4) and the SARS-CoV-2 spike glycoprotein has been recognized as a potential mode for viral entry into the host cell [117,118,119,120]. CD26 exists in two forms: the membrane- anchored (mCD26) and the soluble form (sCD26) [121]. Although SARS-CoV-2 primarily infects the lungs, studies have reported gastrointestinal (GI) invasion of SARS-CoV-2 and the GI tract as a major site of viral infection [122,123]. The SARS-CoV-2 spike (S) glycoprotein binds DMT1 (divalent metal-binding proteins) on epithelial cells in the gastrointestinal mucosa that could result in membrane fusion and virus entry into the cells [117]. A previous study has shown that mCD26 found on duodenal cells in the GI tract competes with the viral spike glycoprotein for binding to DMT1, thus preventing viral entry through gut mucosa, leading to beneficial immune-protective effects [124]. Studies have shown decreased sCD26 levels in people with diabetes, dementia, and elderly individuals [117]. This is in line with the clinical finding of increased susceptibility to the virus as seen in individuals with diabetes and cardiovascular diseases [125].

The dynamic response of the immune system is attributed to protection against several viruses including SARS-CoV-2. However, the emerging variants of the virus hijack and suppress the immune response, causing lethal consequences including multi-organ failure in some patients. Thus, dissecting the interplay between SARS-CoV-2 and the host immune system will significantly enhance our understanding of viral pathogenesis.

### 1.3. Interaction of SARS-CoV-2 with Host RNA Binding Proteins and miRNAs

The process of SARS-CoV-2 infection and its trafficking inside the host cell is complex and requires remodeling of the cellular machinery that results in the disruption of several key events in the host cell, such as splicing and translation [75]. SARS-CoV-2 utilizes host derived proteins and RNAs to facilitate viral replication and the assembly of new virions [126]. Computational study on SARS-CoV-2–host interactions has identified several suppressed antiviral immune response pathways such as Toll-like receptor signaling, IL-17 signaling, hypoxia-inducible factor 1 (HIF-1) signaling, and retinoic acid-inducible gene I (RIG-I) signaling [126]. SARS-CoV-2 modulates the host immune response by suppressing these signaling pathways to support the viral life cycle and propagate infection. Several groups have explored the physical association between viral and host proteins and identified protein–protein interaction networks, revealing several important drug targets for effective antiviral therapies [90,93,127,128]. Recently, Gordon et al. used affinity purification mass spectrometry to identify 332 high-confidence protein–protein interactions between SARS-CoV-2 and human proteins that could be targeted for suppressing viral infection [90]. Given the high mutation rate in the large RNA genome of SARS-CoV-2, recent studies have used a RNA-centric approach to investigate the crosstalk between viral RNA and human RBPs resulting in the altered regulation of host molecular functions. Flynn et al. identified 309 host proteins that interact with SARS-CoV-2 RNA using comprehensive identification of RNA-binding proteins by mass spectrometry (ChIRP-MS) [129]. For instance, the study identified the interaction of viral RNA with Rab GTPase proteins RAB10 and RAB2A, which are essential for subcellular trafficking. Thus, it is most likely that SARS-CoV-2 might exploit specific Rab GTPase in the host cell to promote viral replication. Perturbation of these proteins by CRISPR revealed their essential role in virus-induced cell death [130]. Findings from the study suggest that the vast majority of the SARS-CoV-2-interacting host proteins were related to functions protecting the host cells and therefore are more likely hijacked for viral pathogenesis. [130]. A broad range of host proteins have been found to physically interact with viral RNA, including those that are not necessarily related to viral recognition functions such as TLRs [129]. Interestingly, Flynn et al. compared their formaldehyde-based ChIRP-MS findings to a set of host factors identified by Schmidt et al. that employed UV crosslinking-based RAP-MS [87]. Their analysis revealed an enrichment for additional 199 proteins (64%) by formaldehyde-based ChIRP-MS that were not significant in the RAP-MS dataset. The inherent crosslinking differences between UV and formaldehyde, particularly regarding higher specificity but not necessarily functional interactions of UV compared to formaldehyde crosslinking, accounts for the increased number of ChIRP-MS-identified RBPs in this study [129]. A similar study from Lee et al. used a modified RAP-MS protocol using UV crosslinking in SARS-CoV-2-infected Vero cells. Their study identified 109 host factors that bind directly to SARS-CoV-2 RNA. Transcriptomic analysis and RBP knockdown experiments further identified eight pro-viral RBPs and 17 anti-viral RBPs that regulate viral RNA replication [131]. A recent study by Kamel et al. employed UV crosslinking-based comparative RNA interactome capture (cRIC) and viral RNA interactome capture (vRIC) to study cellular and viral proteins that are altered upon SARS-CoV-2 infection in epithelial human lung cancer cells (Calu-3) [132]. Similar to the observation from Flynn et al, this study also describes the lower efficiency but higher specificity of UV crosslinking compared to formaldehyde crosslinking. This further explains why some proteins linked to viral RNA metabolism (within ORF1a/b) could not be identified by the vRIC approach used in this study. Their analysis revealed that SARS-CoV-2 remodels the cellular RNA-bound proteome (RBPome), including proteins that play a crucial role in viral life cycle, RNA metabolism, and anti-viral response [132]. The study identified 809 proteins enriched with RNA-binding domains, out of which 335 RBPs demonstrated a significant fold change at 24 h post infection [132]. The study also illustrated the functional importance of the identified RBPs based on their prevalence in genome wide screens (RNA interference, CRISPR-Cas9, and haploid line screens) with 36 viruses from previous studies. Their analysis revealed that 67 RBPs detected by vRIC, while 73 upregulated and 51 downregulated RBPs identified by cRIC, were linked to viral infection previously. To further assess the therapeutic value of the identified RBPs, the authors tested five drugs from the drug–gene interaction database and found that two drugs that target HSP90 and IGF2BP1 strongly inhibited viral protein production, suggesting the importance of these proteins as potential anti-viral drug targets. Overall, these studies provide significant insights into the viral–protein RNA interaction in host cells through genome-wide and proteome-wide approaches using crosslinking methods. More in-depth experimental evidence will further bridge the mechanistic understanding of the interaction between viral RNA and host proteins that regulates post-transcriptional regulation and viral life cycle.

Recently Srivastava et al. systematically dissected the dysregulated RBPs during SARS-CoV-2 infection using publicly available genomic datasets [133]. The study revealed that 13 out of 29 SARS-CoV-2-encoded proteins exhibited direct interaction with 51 human RBPs, with an abundant expression in gonadal tissues and immune cells. Several host proteins such as SRSFs, ELAVs, HNRNPs, and PCBPs had a binding site on the SARS-CoV-2 genome. Thus, the study implied that the viral genome could titrate these RBPs and alter the host’s post-transcriptional regulation. Schmidt et al. identified 104 proteins in humans that bind viral RNA using RNA antisense purification and mass spectrometry [87]. The authors identified crucial proteins such as the nucleic acid-binding protein (CNBP) and La-related protein 1 (LARP1) among the viral interacting proteins. The study further illustrated that proteins exhibiting viral interaction were enriched for crucial functions pertaining to viral transcription, translation initiation, non-sense-mediated decay, and protein-targeting to the membrane, suggesting that these functions could be altered during viral pathogenesis [87]. Furthermore, 19 ribosomal proteins and 12 translation factors were found to interact with viral RNA, suggesting their possible involvement during viral protein translation. 

Another study investigated the interaction between 5′ and 3′ UTR of SARS-CoV-2 RNA and host proteins, revealing an enriched expression of proteins involved in RNA metabolism and translation initiation [134]. The study also identified Lysosome-associated membrane protein-2a (Lamp2a) as one of the 5′-UTR interacting proteins. Interestingly, Lamp2a overexpression was found to reduce the viral RNA load in infected cells and vice versa, suggesting an antiviral function of the protein through its interaction with viral 5′ UTR. 

In addition to host RBPs, microRNAs that are small non-coding RNA molecules (18-20 nucleotide) have recently emerged as crucial regulators of gene expression during viral infections [135,136,137]. However, a detailed understanding of the host miRNAs that may regulate SARS-CoV-2 pathogenicity is only beginning to be explored [138,139,140,141]. In general, host cells produce miRNAs during the early stage of viral infection to impede viral replication and translation. However, viruses can bypass the targeted degradation by the host miRNA and host defense response [136]. In a recent study, the authors identified binding sites of several human miRNAs within the SARS-CoV-2 ORFs and 5′ and 3′ UTRs [142]. Strikingly, some of these miRNAs’ binding sites were also identified in the 3′ UTRs of host mRNA transcripts encoding viral entry proteins such as ACE2, TMPRSS2, and IFN-α. Overall, these studies suggest that miRNAs binding to the SARS-CoV-2 genome could significantly impact the viral pathogenesis and alter host regulatory mechanisms. Table 1 summarizes the various studies that reported the host RNA-binding proteins and microRNAs interactions across the SARS-CoV-2 viral genome, using both in silico and experimental techniques.

Another machine learning-based study predicted that miRNAs could modulate SARS-CoV-2 infection by regulating the expression of the host genes involved in replication, transcription, defense response, and signaling pathways, including EGFR and Wnt [140]. A previous study from our group identified 22 miRNAs that could potentially be sequestered on the SARS-CoV-2 genome. Interestingly, immune cells were found to have an abundant expression of these miRNAs, indicating that viral interaction with miRNAs in immune cells could modulate a host immune response [133]. Firstly, this could disrupt the normal homeostasis in host cells due to the depletion of miRNAs that would upregulate the expression of host mRNAs that were typically targeted by these miRNAs. Secondly, titration of host miRNAs on the viral genome could enhance viral replication and suppress the host immune response [143]. Human miRNAs have been shown to target various viral mRNA, including those encoding for structural proteins (Spike, envelope, membrane, and nucleocapsid) as well as the non-structural protein transcripts (ORF1ab, 3a, 6, 7, 8, and 10), as summarized in a previous study [140]. With the increasing evidence for the involvement of host miRNAs in the pathogenesis of SARS-CoV-2 infection, focused studies are required to validate their interaction with the viral genome that would further create opportunities for miRNA-based antiviral therapies.

### 1.4. Impact of Mutations on SARS-CoV-2 Proteins

SARS-CoV-2 rapidly evolves in humans and therefore it produces several variants that affect the host immune response to viral pathogenesis [144,145]. The virus protein structure is vital for viral function and any change to this could affect immune system interactions and lead to host proteins with altered functions. According to a comprehensive SARS-CoV-2 secondary structure study by Huston et al., 97% of the SARS-CoV-2 genome has not been structurally examined, making drug-targeted therapeutics challenging [146]. Therefore, it is imperative to investigate the mutations and their associated patterns on the SARS-CoV-2 genome. Recently, genomic mutation analysis of SARS-CoV-2 has demonstrated the prevalence of P4715L and T265I mutations in 40% and 21% of the SARS-CoV-2 sequences, respectively [144].

Four different types of mutations were detected in the virus: synonymous, nonsynonymous, insertion, and deletion [144]. Analysis of the viral spike protein demonstrated no insertions, 28 deletions, and 240 non-synonymous and 670 synonymous mutations [144]. The mutation that stood out was D614G [144]. Analyzing the RBD (receptor-binding domain), the study found 53 non-synonymous and 46 synonymous mutations. A total of 69 mutations that can change protein secondary structure were identified, with four mutations (S477G, P479L, V483A, and F486L) having the potential to change the protein secondary structure in the RBD region alone [144]. The study also revealed that out of the 68 mutations detected, only three mutations (V483A, F486L, and G485R) were inferred to have the potential to change solvent accessibility in the RBD region [144]. Overall, 48 mutations were found to potentially alter the structure and solvent accessibility, with the most significant mutation, D614G, not having either ability. Notably, the spike protein exhibited several variants, including D614G as the most considerable mutation [147,148].

Furthermore, eight non-synonymous mutations were identified in the spike protein with an ability to change its amino acid sequence, including the most significant variants N354D, D364Y, and V367F [144,149]. Mutations in the spike protein have been suggested to lead to shape changes which would then affect antigenicity [149]. In the same manner, recurrent mutations have also been found in specific regions within the spike protein [150]. The mutation of spike protein codon 614 from aspartate to glycine (D614G) has been abundantly associated with the SARS-CoV-2 genome [151,152,153]. As D614G occurs so frequently in the dataset, one could infer that the modification attributes to a more transmissible virus. However, the impact of D614G on virulence or vaccine efficacy needs to be further evaluated. 

Nucleocapsid proteins are an integral component of the viral structure and therefore mutations in the nucleocapsid are likely to affect the host anti-viral response [148]. Nguyen et al. showed that while there are no insertion mutations in the nucleocapsid protein, there are a total of 15 mutations that have the potential to alter the protein structure and solvent accessibility [144]. The study uncovered 156 non-synonymous mutations with two significant mutations, R203K and G204R [144]. Recent genotyping analysis of SARS-CoV-2 revealed specific mutations to be predominant in the current pandemic, including G28881A, G28882A, and G28883C in the nucleocapsid protein [148]. Mutation R203K (arginine to lysine) was associated with the structural change occurring in the nucleocapsid protein; the researchers used the SCRATCH-1D software for the structural analysis [144]. Importantly, R203K mutation is extensively studied for its therapeutic value because of its frequent occurrence in SARS-CoV-2 genomes [154,155,156].

Recently, Koyama et al. utilized the epitope information to investigate the viral drift variants and their associated mutations. Their study predicted that 12 different mutations in the SARS-CoV-2 spike, nucleocapsid, and membrane proteins might contribute to the generation of drift variants [147].

The envelope protein, critical for viral packaging, has been recognized as an important target for vaccine development. Therefore, investigating mutations on the envelope protein is essential for understanding viral pathology. A recent study used SCRATCH-1D software to predict the likelihood of mutation-related structural changes in the SARS-CoV-2 genome. The study revealed five non-synonymous mutations in the envelope protein that can alter the protein structure (S68C, S68F, P71L, D72Y, and L73F). Four mutations were found to potentially shift the solvent accessibility (L37H, L37R, D72Y, and L73F), among which D72Y and L73F were demonstrated to have the ability to change both the protein structure and the solvent accessibility [144]. 

ORF1ab, the largest gene in the viral genome with an extensively folded RNA structure, encodes polyproteins pp1a and pp1B that are cleaved into non-structural proteins (Nsps) critical for viral replication and translation, making it an important drug target for anti-viral therapeutics [146]. The large size of the ORF1ab protein has been attributed to the presence of several mutations [19,144]. Mutation analysis of ORF1ab demonstrated two insertions (3603F and 7041F), 48 distinct deletions, and 1067 non-synonymous mutations [144]. A study focusing on mutations on the SARS-CoV-2 genome identified 198 recurrent mutations, 80% of which resulted in non-synonymous changes in the respective protein structures [150]. The authors from this study found >15 recurring mutations in the ORF1ab encoding for Nsp6, Nsp11, Nsp13, and the spike protein. The findings from this study suggest recurrent mutations as possible modes of viral adaptation in the host cell.

Similar to other structural proteins, the membrane protein has also been extensively studied for the presence of mutations. Thirty-seven non-synonymous mutations were found in the membrane protein and no insertion or deletion mutations were observed in a previous study [144]. Furthermore, 10 mutations exhibited the potential to change the protein secondary structure (C64F, A69S, A69V, V70F, N113B, R158L, V170I, D190N, D209Y, and S214I) [144]. At the same time, six mutations in the membrane protein showed the ability to change the solvent accessibility [144].

**Figure 2:**
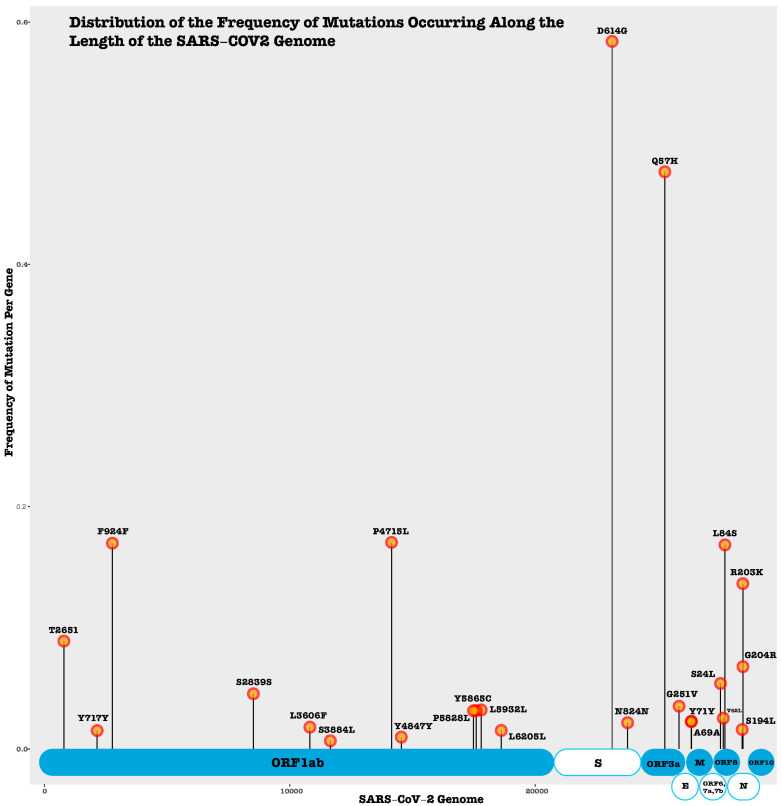
Mutations (synonymous, non-synonymous, deletion, and insertion) occurring across the SARS-CoV-2 genome. Only modifications that appear over 100 times in the dataset are in the figure. The frequency of each mutation by gene was calculated and displayed in the plot. Data for the plot can be found in the Supplementary Material of this publication. Genomic modifications and changes in the protein secondary structure and solvent accessibility of SARS-CoV-2 (COVID-19 virus) [144].

Among the non-structural protein encoding genes, ORF3a plays a crucial role in viral replication and pathogenesis, exhibiting one insertion, six deletions, and 125 non-synonymous mutations including Q57H and G251V, the significant mutations [144]. ORF6 revealed two insertions, nine deletions, and twenty-three non-synonymous mutations [144]. The study showed that ORF7a had no insertions, 15 deletions, and 32 non-synonymous mutations [144]. In comparison, ORF7b showed no insertion or deletion mutations and 10 distinct non-synonymous mutations [144]. ORF8 was found to have no deletions, four insertions, and fifty non-synonymous mutations [144]. Notably, ORF10 demonstrated no insertions or deletions and only eight non-synonymous mutations [144]. In order to provide further insights into the frequency of various mutations (synonymous, non-synonymous, deletion, and insertion) occurring across the length of the SARS-CoV-2 genome, we utilized previously published mutation data from Nguyen et al. [144]. Our analysis demonstrated that 58% of the mutation data was contributed by four specific mutations including D614G, Q57H, P4715L, and F924F (Figure 2). The nucleocapsid protein exhibited the highest number of mutations leading to structural changes. The most recurring mutations from the dataset were D614G, Q57H, F924F, P4715L, L84S R203K, D614G, P4715L, Q57H, and L84S [144]. The functions of these mutations are not fully understood; however, they could potentially be implicated with a higher transmission rate in the evolving strains of the virus. Detecting mutations that cause higher virulence and transmissibility is challenging because it is most likely a combination of mutations that yield a more pathogenic strain and there is limited clinical metadata associated with patients who have also been well genotyped for the specific strains of SARS-CoV2 in several parts of the world.

Several lines of evidence have demonstrated that mutations in the SARS-CoV-2 genome have led to the emergence of pathogenic strains that demonstrate reduced vaccine efficacy, resulting in breakthrough infection after vaccination [157,158,159,160]. A recent study reported breakthrough infection in two women out of 417 individuals that were observed two weeks after the second dose of the BNT162b2 (Pfizer-BioNTech) or mRNA-1273 (Moderna) vaccine [157]. Sequencing of the viral variants from both women revealed the presence of three mutations including D641G, T95I, and del142-144 that may be responsible for the breakthrough infection. Vaccines from AstraZeneca, Johnson & Johnson, and Novartis have also been reported to exhibit reduced efficacy against the B.1.351 strain [158,160]. Weissman et al. recently reported that the D614G mutation on the spike protein of the virus could contribute to neutralization escape by vaccine and thus reduce the vaccine efficacy [161]. These observations indicate that vaccinated individuals may still be susceptible to the risk of reinfection and severe illness due to the emergence of more pathogenic strains of the virus. The widespread infection rate and enhanced transmissibility of the virus have posed a tremendous challenge for clinicians to design vaccines that can effectively block these pathogenic mutations in variants of concern.

SARS-CoV-2 mutations have been associated with a higher mortality rate than the original strains and have been summarized in a previous review [162]. For instance, the viral ORF 3a protein mutation was associated with several modifications in the viral structure, such as the loss of the leucine zipper motif and B cell epitope loss that could further contribute to the development of more pathogenic characteristics in the evolving strain [163]. Mutation in the viral spike protein (G614) was recently shown to destabilize the spike protein that attributed to an increased fatality rate [164]. Overall, the mutations in the viral genome have significantly enhanced the pathogenic characteristics in the new strains with higher morbidity and mortality in patients.

### 1.5. Databases and Software Resources for the SARS-CoV-2 Genome

Human–pathogen interactions (HPI) describe the molecular crosstalk between native host and invading pathogen molecules. Several different types of HPI resources are currently available, including those dedicated to studying protein–protein interactions (PPI), protein–RNA interactions (PRI), and RNA–RNA interactions (RRI). A detailed understanding of SARS-CoV-2 HPI helps researchers describe mechanisms for viral invasion, replication, and the host response.

Table 2 shows eight publicly available databases containing at least one of the SARS-CoV-2 HPI types. These resources catalog annotated and predicted PPI; IntAct also includes data for PRI. Several methods are used for assembling such data, with some databases providing only curated HPI (SARS-3D and BIOGRID); others have predicted HPI or user-submitted data (VirHostNet). Table 3 shows six databases with viral HPI data. While they do not yet contain any SARS-CoV-2 information, these resources are good candidates for future HPI, especially as the volume of validated SARS-CoV-2 HPI continues to grow. Most of these databases predominantly focus on viral PPI and include both predicted and annotated data. The HPIDB 3.0 database also documents RRI interactions.

HPI is necessary for the development of effective treatments and vaccines against SARS-CoV-2. Several public databases catalog important SARS-CoV-2 PPI and PRI and present them in useful formats for download and analysis. Other databases are also poised to support SARS-CoV-2 HPI data. There is a notable paucity of databases containing PRI or RRI data; however, we anticipate that new information from emerging studies will become available and an opportunity arises for existing or new databases to capture and integrate such datasets.

## 2. Conclusions

This review summarizes the recent discoveries in the interaction of SARS-CoV-2 with various host cellular components and the immune system. Among the various interactions, ACE2 and TMPRSS2 proteins have been recognized as the major host proteins that are exploited by the virus for its entry into the host cell [165,166]. Other host proteins such as BRD2 and BRD4 were shown to interact with viral envelope proteins, thereby disrupting the histone acetylation, resulting in the altered transcriptional regulation of host genes [90,167]. Furthermore, host RNAs such as small nuclear RNA (snRNA), 18s rRNA, and 7SL RNA, which are vital components of the signal recognition particle (SRP), were found to interact with various non-structural proteins (NSP) of the SARS-CoV-2 [75]. This interaction was shown to disrupt the SRP regulated protein synthesis that could potentially suppress the host defense. Several viral components, including various non-structural proteins, exhibited interaction with host RNAs. For instance, the interaction between Nsp1 and host 18s rRNA was shown to impede the translation of host mRNA in SARS-CoV-2-infected cells [168]. Overall, the interactions between SARS-CoV-2 and host cellular components could modulate vital functions in the host cell, leading to an impaired immune response.

The review also emphasizes the interaction of SARS-CoV-2 with various components of host immune signaling pathways, including the IFN pathway (Nsp13, Nsp15, Orf9b), NF-kB pathway (Nsp13 and Orf9c), and IL17 signaling pathway (Orf8) [87,89,90]. These interactions have demonstrated an altered immune response, such as reduced pro-inflammatory cytokine secretion leading to an altered host immune response. We also present emerging evidence from multiple studies showcasing the crosstalk of SARS-CoV-2 with host RNA-binding proteins and miRNAs that could potentially impact the host anti-viral response (Table 1). Using mass spectrometry, 332 protein–protein interactions were identified between SARS-CoV-2 and host proteins [90]. Among the interacting proteins, several of the proteins were found to have an essential role in maintaining cellular homeostasis. For instance, Rab GTPases (RAB10 and RAB2A) vital for subcellular trafficking were found to interact with viral proteins that could be exploited for viral pathogenesis.

We also provide insights into the various mutations identified across the SARS-CoV-2 genome and their prevalence in various structural and non-structural components of the virus. Mutations occurring across the SARS-CoV-2 genome could lead to the development of a new strain that can bypass the current therapeutic strategies for the treatment of COVID-19. Multiple variants of SARS-CoV-2 resulting from the mutations are increasingly being identified globally. As defined by the WHO, variants of concern (VOC) are Alpha, Beta, Gamma, and Delta, while variants of interest (VOI) are Eta, Iota, Kappa, and Lambda. VOC has been attributed with evidence for a higher transmission rate, reduced susceptibility to vaccines, and increased death rate. In comparison, VOI is suspected to be more contagious than the original strain and requires further investigation to establish its virulence.

The Center for Disease Control and Prevention (CDC), in collaboration with public health agencies, has recognized six SARS-CoV-2 variants of concern, including (i) B.1.1.7 (U.K or Alpha variant), identified in the U.S. in December 2020; (ii) B.1.351 (South African or Beta variant); (iii) P.1 (Brazilian or Gamma variant); (iv) B.1.617.2 (Indian or Delta variant), identified in India at the end of January 2021; (v) B.1.427 and B.1.429 (California, U.S. variant), identified in February 2021; and (vi) C.37 (South America or lambda variant) [26,27,28,29,169,170]. The Brazilian variant P.1 was recorded to exhibit 17 unique mutations, of which three were found in the viral spike protein [171,172,173]. The newly identified strains of SARS-CoV-2 appear to be more infectious and transmitted readily compared to the older variants. The new variants might also impact the effectiveness of vaccines and antiviral drugs currently used to treat COVID-19. Moreover, the newly mutated strains are also suspected of skipping detection by viral diagnostic tests such as RT-PCR, making it harder to identify the infectious strain. So far, clinical studies have suggested the efficacy of available vaccines; however, a closer investigation is required to study the impact of new and emerging variants on vaccine-induced immunity in the host or vaccine resistance.

We present a repertoire of publicly available databases and software that enable users to study the crosstalk between host cell components and viral genomes (Table 2). With the emergence of novel variants, there is a need to develop updated databases and resources for the identification and characterization of evolving viruses to enable researchers to keep track of mutations across the viral genomes. This will further enhance our understanding of viral evolution and its impact on host cellular components. This necessitates the importance of tracking mutations and the resulting strains of SARS-CoV-2 across the world. Further studies in this direction are required to explore new variants’ functions and enable the development of effective anti-viral therapeutics for evolving infectious strains.

## Figures and Tables

**Table 1:** Interaction between SARS-CoV-2, human RBPs, and microRNAs.

Interactions between SARS-CoV-2, Human RBPs, and miRNAs	Technique Used	References
332 SARS-CoV2–host protein–protein interaction	AP-MS	[90]
309 host proteins interaction with SARS-CoV-2 RNA	ChIRP-MS	[129]
Host RAB2A, RAB7A, and RAB10 interaction with both viral RNA and protein	CRISPR Cas-9-based perturbation	[130]
25 human RBPs targeting SARS-CoV-2 viral RNA	RBP motif-based in silico prediction	[133]
104 human proteins directly and specifically bind to SARS-CoV-2 RNAs	RAP-MS	[87]
288 host miRNAs predicted to bind SARS-CoV-2 (ORF1ab, N, S, 5′-UTR, and 3′-UTR)	Bioinformatic prediction algorithms and miRNA profiling	[142]
479 human miRNAs could target various SARS-CoV-2 genes (S, E, M, N, Orf 1ab, 3a, 6, 7, 8, and 10)	Machine learning-based miRNA prediction	[140]
22 miRNAs could bind throughout the length of the SARS-CoV-2 viral genome	Computational approach using FIMO	[133]

**Table 2:** Publicly available databases containing at least one of the SARS-CoV-2 HPI types. These databases were last accessed in June 2021.

Database	Interaction(s)	URL	Description
SARS-3D	Protein–protein	http://sars3d.com (accessed on 30 June 2021)	3D protein models predicted using genome data
VirHostNet	Protein–protein	http://virhostnet.prabi.fr (accessed on 30 June 2021)	Interactions between SARS-CoV-2 and human proteins
BioGRID (curated dataset)	Protein–protein	https://thebiogrid.org/project/3 (accessed on 30 June 2021)	Curated coronavirus dataset with 22,223 interactions over 110 proteins
IntAct	Protein–protein and protein–RNA	https://www.ebi.ac.uk/intact/query/annot:%22dataset:coronavirus%22 (accessed on 30 June 2021)	Over 4400 binarized SARS-CoV-2–human molecular interactions
Human Proteome Atlas	Protein–protein	https://www.proteinatlas.org (accessed on 30 June 2021)	Summary of tissue and cell expression patterns of human proteins interacting with SARS-CoV-2
Intomics	Protein–protein	https://www.intomics.com/covid19/?utm_source=intomics&utm_medium=linkedin&utm_campaign=covid19 (accessed on 30 June 2021)	PPI network based on transcriptional response in human SARS-CoV-2-infected cells
Protein Data Bank	Protein–protein	https://www.rcsb.org/news?year=2020&article=5e74d55d2d410731e9944f52&feature=true (accessed on 30 June 2021)	Protein–protein complex crystal structures (i.e., S—ACE2 complex)
STRING-DB	Protein–protein	https://string-db.org/cgi/covid.pl (accessed on 30 June 2021)	Protein–protein interaction network with 332 virus-interacting human proteins

**Table 3:** General resources and databases with viral HPI data. These databases were last accessed in June 2021.

Database	Interaction Type(s)	URL	Description
Viruses.STRING	Protein–protein	http://viruses.string-db.org/ (accessed on 30 June 2021)	Catalog of virus–host PPI
HVIDB	Protein–protein	http://zzdlab.com/hvidb/ (accessed on 30 June 2021)	Annotated human–virus PPI
P-HIPSTer	Protein–protein	http://phipster.org/ (accessed on 30 June 2021)	PPI predicted from PDB structure data
VirusMentha	Protein–protein	https://virusmentha.uniroma2.it/ (accessed on 30 June 2021)	Regularly updated PPI
Virus MINT	Protein–protein	https://maayanlab.cloud/Harmonizome/resource/Virus+MINT (accessed on 30 June 2021)	5000 PPI covering 110 viral strains
HPIDB 3.0	RNA–RNA, protein–protein	https://hpidb.igbb.msstate.edu/ (accessed on 30 June 2021)	69,787 annotated and predicted interactions

## Data Availability

The study analyzed public dataset available in the bioRxiv repository, https://www.biorxiv.org/content/10.1101/2020.07.10.171769v2.supplementary-material to generate Figure 2.

## Supplementary Materials

The following are available online at https://www.biorxiv.org/content/10.1101/2020.07.10.171769v2.supplementary-material.
